# Supplementary material for: iSTTC: A robust method for accurate estimation of intrinsic neural timescales from single-unit recordings
Source: PLoS Comput Biol. 2026 Mar 16;22(3):e1013385. doi: 10.1371/journal.pcbi.1013385 (PMC13012623; doi:10.1371/journal.pcbi.1013385)
Supplement: Attachment — Submitted filename: response_to_reviewers.docx [file pcbi.1013385.s015.docx]

# Note for the editor:

# We detected a minor bug in the plotting script for Figure 5B,C. We corrected it and regenerated the figure. Only the values changed slightly; all the underlying conclusions were unaffected.

# Reviewers' comments:

Reviewer's Responses to Questions

## R1

Reviewer #1: In this manuscript, the authors propose a new method (iSTTC) to estimate the auto-correlation time from spiking trains of single neurons. This auto-correlation time, frequently called 'intrinsic timescale' (IT), has been of continuing interest because it varies systematically across brain regions and tends to be correlated with behavior. Accordingly, the method is potentially relevant for the neuroscience community. Furthermore, it is, to the best of my knowledge, novel.

The manuscript starts with a concise but nicely executed summary of the ongoing interest in intrinsic timescales. In the results, the authors start with a brief description of their method (2.1), then they compare it to existing methods on simulated data (2.2-2.5), and finally they apply their method to mouse data from the Allen Institute (2.6). Structure, writing, and figures are mostly clear and easy to follow. Commendably, the code is publicly accessible and clearly organized; it runs but I did not check the implementation in detail.

The main result is that the proposed method outperforms existing ones. While the authors demonstrate this for two commonly used approaches, my main criticism is that they did not compare it to the method by Zeraati, Engel, and Levina (ref 25 in the manuscript). The latter already addresses several of the problems discussed in the third paragraph of the introduction, accordingly it provides a much more competitive baseline. Taking also the remarks below into account, I believe that the manuscript needs a major revision.

We thank the reviewer for the thoughtful comments and the opportunity to clarify our results and their implications. Overall, the reviewers’ suggestions substantially improved the manuscript.

Major remarks:
1 * The dominant effect in the Figures seems to be that all methods have an estimation error of at least 10 (often 100, sometimes 1000) percent. Furthermore, the total error is always much larger than the difference between iSTTC and the compared method. Naively, this seems to suggest that there is currently no method to reliably estimate the IT - which makes it even more important to compare to ref. 25.

We agree that estimating intrinsic timescales remains a difficult problem and that all existing approaches, including ours, retain substantial error. The goal of this work is not to claim that iSTTC yields perfectly accurate IT estimates, but to show that improving the ACF‐estimation step systematically reduces REEs relative to current practices.

To address the request regarding ref. 25, we have now added a dedicated section (2.7: “iSTTC and aABC are designed for different use cases” and 7.6: “Intrinsic timescale estimation with adaptive Approximate Bayesian Computations”) and a new Supplementary Figure (Supp. Figure 11) directly comparing iSTTC to adaptive Approximate Bayesian Computation (aABC). As detailed in that section, aABC optimizes step 2 of the IT estimation pipeline: obtaining IT from the ACF. Conversely, iSTTC improves step 1 by providing a more robust ACF-like estimate. Moreover, the computational cost of aABC makes it unsuitable for large-scale single unit datasets and long unsegmented spiking data. As we note in the manuscript, applying aABC to large single-unit datasets is in principle possible, but its computation time makes such an application highly impractical. This is also consistent with prior work: the method is typically used on population activity (pseudo - MUA) and epoched data, rather than on long single-unit recordings ([Zeraati et al. 2023](https://paperpile.com/c/RhYcRp/AyVn)). Lastly, when analysing long SUA recordings, the authors of aABC themselves relied on direct exponential fitting rather than applying aABC (Shi et al. 2025). This is because for long time series the bias in autocorrelation estimates is negligible. Thus, iSTTC and aABC optimize different steps of IT estimation and are designed for largely non-overlapping use-cases.

Nonetheless, we have compared aABC to iSTTC using a subset of the dataset of Figure 5. iSTTC applied on unsegmented data remains more accurate and inclusive than aABC. On epoched data, aABC outperforms iSTTC and PearsonR primarily by suppressing very large overestimates, but at the price of longer runtimes (median 212 s vs. 0.038 s per unit, >5,000-fold difference) and a non-negligible rejection rate of units for which the algorithm fails to produce an IT estimate.

Taken together, these new analyses suggest that, while no single method currently eliminates IT estimation error, iSTTC and aABC improve different stages and use-cases of the IT estimation pipeline and are therefore complementary rather than competing. We now explicitly state that combining an iSTTC-derived ACF-like (by adding it as a summary statistic to abcTau toolbox) with aABC-based fitting could, in principle, further improve IT estimation, although this lies beyond the scope of the present work.

2 * It is not clear to me why spike trains can be concatenated in iSTTC (claimed several times to be "a key advantage"), and I did not find an explanation for this in the Methods.

As suggested, we have now clarified why and how trial concatenation is performed in iSTTC, and how the method avoids artefacts introduced by padding.

Because iSTTC compares spike timing within a continuous time window, directly concatenating trials would create spurious correlations across trial boundaries. To prevent this, we concatenate trials with zero-padding of sufficient length (equal to or greater than the trial duration), ensuring that spikes from adjacent trials do not influence the iSTTC calculation.

The iSTTC implementation for trials differs from that for unsegmented data only in how the “T” term is computed, which ensures that zero-padding does not affect the output. Since T is defined as the proportion of total signal length that lies within ± Δt of any spike, it depends on the effective signal duration. Zero-padding artificially increases the signal length, and computing T on the padded signal would therefore bias the STTC. To avoid this artefact, we compute T within each original trial before padding, using the true trial duration, and then averaging these values across trials. This provides a fixed and unbiased T value for each time lag and ensures that changes in the autocorrelation across lags reflect genuine temporal structure rather than changes in the effective signal length introduced by padding. As the calculation of the P term is unaffected by signal length, we compute P on the concatenated signal.

We have expanded the method description in Section 2.1 (lines 110 - 127), added a new panel (Figure 2C) illustrating the application of iSTTC to trials, and included a pseudocode description of the algorithm in Section 7.2.4.

3 * Regarding the ground-truth model: Is the auto-correlation function for this model exponential? And is tau = tau_kernel / (1 - alpha) the time scale of this exponential (the parameters seem to be set based on this assumption)?

We clearly specified that for a univariate Hawkes process with an exponential kernel, the autocorrelation function is an exponential decay ([Hawkes 1971](https://paperpile.com/c/OI7Jze/V0A7), [Hawkes and Oakes 1974](https://paperpile.com/c/OI7Jze/5GKB)). The decay time constant of this exponential ACF is given by tau_ACF = tau_kernel / (1 – alpha). Based on this relationship, we set the kernel time constant in our simulations to tau_kernel = tau × (1 – alpha), which ensures that the resulting spike trains have an intrinsic timescale equal to the target value tau. This value serves as the ground-truth timescale that our estimators aim to recover.

We have added this information to the synthetic dataset description in Section 7.1.1 (521 - 527). We also included Supp. Figures 1, 5, and 9, illustrating the ACF traces and explicitly showing the ground-truth ACF.

4 * Given that the manuscript introduces the method it would be nice to provide a detailed explanation as well as a graphical overview (similar to Supp. Sec. 7.2.2. and Supp. Figure 1) in the main text. Conversely, the description in 2.1 is too brief to understand the method.

To address the reviewer’ comment, we improved the written and graphical description of iSTTC. We followed the reviewer’s suggestion by expanding the method description in Section 2.1 (lines 93 – 127). In addition, we moved Supp. Figure 1 into the main text as the new Figure 2, expanded the iSTTC schematic in Figure 2B, and added a new panel (Figure 2C) illustrating the application of iSTTC to trials. We also included pseudocode descriptions of algorithms in Section 7.2.2 (iSTTC) and Section 7.2.4 (iSTTC for trials).

Minor remarks:
* "Ogata’s thinning algorithm" needs a reference.

We have added two references (line 513):

Ogata, Y. (1981). On Lewis’ simulation method for point processes. *IEEE Transactions on Information Theory*, *27*(1), 23–31. <https://doi.org/10.1109/tit.1981.1056305>

Ogata, Yosihiko. (1988). Statistical models for earthquake occurrences and residual analysis for point processes. *Journal of the American Statistical Association*, *83*(401), 9. https://doi.org/10.2307/2288914

* Eq. (2) seems to contain typos: it should be g(Delta) instead of gDelta, there is a trailing bracket, there should be a comma before Delta ge 0.

We fixed the typos.

* Below eq (2) I believe tau -> tau_kernel below eq (2).

We changed tau to tau_kernel.

* Missing dependencies in the repository: jupyter, matplotlib, statsmodels, seaborn

We have added missing dependencies to the .yaml file.

* The link to gin for the synthetic dataset does not work (404 error).

Our GIN repository was not set to public, and we have now corrected this.

* In Fig. 6: should "local variance" be "local variation"?

We changed “local variance” to “local variation”.

## R2

Reviewer #2: This manuscript introduces intrinsic Spike Time Tiling Coefficient (iSTTC), an extension of the spike-time tiling coefficient designed to provide more robust and inclusive estimates of intrinsic neural timescales (INTs) from single-unit recordings. The study is timely because INTs are increasingly recognized as fundamental markers of neural computation and cognition, yet current approaches remain limited by strict inclusion criteria, biases in trial-based data, and instability in the presence of sparse firing. By introducing a method that works well across both continuous and epoched recordings, the authors make an important methodological contribution. The approach is mostly solid, combining synthetic datasets with known ground truth and large-scale Neuropixels recordings, and the authors also demonstrate improvements in inclusion rates, reliability, and robustness. In my opinion, this is a solid contribution that may help future studies of temporal integration in neural systems.

We thank the reviewer for the thoughtful comments and the opportunity to clarify our results and their implications. Overall, the reviewers’ suggestions substantially improved the manuscript.

Major Points

1. A fundamental, yet only tacitly acknowledged, limitation in the field is the absence of a definitive ground truth for identifying interneurons (INTs) in experimental data. While synthetic models permit validation, this is inherently impossible for in vivo recordings. The manuscript proceeds without directly addressing this core issue, presenting results as if a ground truth were available. We strongly recommend that the Discussion explicitly confronts this limitation, highlighting the shortcomings and possible ways to mitigate them.

We thank the reviewer for raising this important point. We assume that, in this context, the reviewer intended to refer to intrinsic timescales (ITs) rather than interneurons (INTs), and we respond accordingly. We fully agree that the absence of ground-truth ITs in experimental data is a fundamental limitation of the field and this is precisely why the core of our benchmarking relies on synthetic data. When operating on experimental data, we agree that direct validation against ground truth is not possible. For this reason, our comparisons mainly focused on other properties of IT estimators that are accessible experimentally, such as robustness of IT estimates as a function of segment length and number of trials, inclusivity of single units, and a comparison of IT estimates on epoched versus unsegmented signals. When using IT estimates from unsegmented data as ground truth (something that we think is justified in light of the much greater accuracy of these approaches on synthetic data), we now explicitly refer to them as “pseudo-REE”. We also agree that this limitation should be stated more clearly. In the revised manuscript, we have renamed the relevant Result subsection (lines 381 - 382), added an explanatory sentence on the definition of pseudo-REE (lines 404 - 407), replaced REE with pseudo-REE in Section 2.8, and explicitly discuss this limitation in the Discussion (lines 456 - 459).

2. The enhanced inclusion rates provided by iSTTC are a valuable advancement. However, it is equally important to consider the neurons that remain excluded. It is unclear whether these units constitute a distinct biological subclass (e.g., neurons with extremely low firing rates or high irregularity), whose systematic omission could bias population-level inferences. A deeper discussion is needed on whether these neurons should be accounted for and what the biological implications of their inclusion or exclusion truly are.

We fully agree that excluding single units from IT estimation can introduce a non-trivial and often underappreciated source of bias. This concern was one of the main motivations for developing iSTTC. In many published studies, IT estimates from SUA are based on relatively strict inclusion criteria (e.g. minimum firing rates), which can lead to worrying rejection rates of up to ~40-60% (see figure below). In contrast, iSTTC does not require such firing-rate thresholds, because it performs particularly well in low firing-rate regimes (Fig. 3C). Consistent with this, in our experimental dataset iSTTC shows no failed fits and all rejected units arise solely from negative R2 values in the exponential fit (Supp. Figure 14A).

We agree with the reviewer that, in future experimental work, it will be important to investigate whether these excluded units correspond to specific cell types, layers or circuit motifs, as this would deepen the biological interpretation of IT estimates. The present manuscript focuses on basic physiological descriptors and shows that rejected units tend to have lower firing rates and lower local variation (Supp. Figure 14B). A more detailed cell-type level analysis lies beyond the scope of this work. However, we have now added a sentence in the Discussion explicitly acknowledging this limitation and pointing out that the potential biological specificity of excluded units is an important question for future studies (lines 494 - 497).

Revision Figure 1. Bar plot displaying the % of rejected units in five recent papers estimating ITs from SUA.

3. The manuscript's comparative analysis is currently limited to ACF and Pearson R. To more fully establish the utility of iSTTC, we recommend contextualizing it against other prominent methods for assessing temporal structure. These include, but are not limited to, the autocorrelation window, detrended fluctuation analysis, Hurst exponent, and measures of spectral slope. Even a conceptual discussion of iSTTC's performance relative to these approaches would help clarify its distinct advantages and limitations within the existing methodological ecosystem.

To address the reviewer’ comment, we added a new analysis and Supplementary Figure (Supp. Figure 11) directly comparing iSTTC to adaptive Approximate Bayesian Computation (aABC), a recently proposed Bayesian framework for IT estimation.

By contrast, the methods explicitly mentioned by the reviewer (autocorrelation window, detrended fluctuation analysis, Hurst exponent, spectral slope) are less straightforward to compare to iSTTC. Several are typically applied to continuous extracellular signals such as LFP or EEG rather than to single-unit spike trains, and many do not yield an IT in physical time units but instead return scale-free exponents.

In fact, we fully share the reviewer’s concern that unifying these different measures is an important goal for the field. This question was a central theme of a recent symposium we organized (4th talk in the workshop “Same words, different worlds: Conceptual consistency in systems neuroscience”, Bernstein Conference 2025). While crucial, a systematic reconciliation of these metrics goes beyond the scope of the present manuscript and would require its own dedicated study. We now explicitly acknowledge this point in the Introduction and refer interested readers to an excellent upcoming review that focuses precisely on this topic ([Zeraati et al. 2024](https://paperpile.com/c/RhYcRp/QmJQ)) (lines 5 - 7).

4. The reliance on a fixed 50 ms lag parameter lacks rigorous justification. The manuscript would be significantly strengthened by a systematic exploration of how iSTTC's performance and the resulting inclusion rates vary across a spectrum of lag values. Demonstrating robustness across timescales (e.g., from fine 10 ms to coarser 100 ms windows) is critical for validating the method and interpreting the biological meaning of the selected integration window.

We agree with the reviewer that relying on a single parameter lag was an arbitrary choice. To address this concern, we have included an additional figure (Supp. Figure 8) and added a new panel (C) to Supp. Figure 10 that discusses in depth how the bin size choice affects IT estimation. For ACF and PearsonR, which operate on binned data, we varied the bin_size parameter using values of 10, 40, 50, 60, and 100 ms. For iSTTC, we used the same set of values as the lag_shift parameter. We show that the choice of bin_size (or lag_shift for iSTTC) affects the number of rejected units, with the highest rejection rates occurring at the smallest bin size/lag_shift. Bin size/lag_shift also influences the estimated intrinsic timescale, although the effect is inconsistent across bin sizes/lag_shifts. Relative to our reference value of 50 ms, a 10 ms window introduces a clear negative bias, 40 ms shows no detectable effect, 60 ms yields a small positive shift in unsegmented data, and 100 ms produces small deviation in the trial-based case. These patterns are highly consistent across methods, indicating that the variability reflects general properties of ACF-based estimation rather than method-specific behavior. We added a new supplementary figure (Supp. Figure 8), a new panel (C) to Supp. Figure 10, included a new section in the results (2.5 Sensitivity of IT estimates to hyperparameter choices), and extended section 2.6 (Lines 327 - 332).

5. While the experimental validation may effectively recapitulate the known hierarchy of timescales, the results should present this in a more direct form with the regions ordered in the x-axis by the hierarchucal index. Moreover, the Discussion could more deeply integrate this result with the anatomical and physiological principles that are thought to underlie it, especially given contradictory reports in the literature.

Siegle JH, Jia X, Durand S, Gale S, Bennett C, Graddis N, et al. Survey of spiking in the mouse visual system reveals functional hierarchy. Nature. 2021;592(7852):86–92.

Piasini E, Soltuzu L, Muratore P, Caramellino R, Vinken K, Op de Beeck H, et al. Temporal stability of stimulus representation increases along rodent visual cortical hierarchies. Nature Communications. 2021;12(1):4448.

This contradiction has also been extended more broaddly in the literaure in which peripheral regions have been proposed to be closer to criticality

https://www.pnas.org/doi/full/10.1073/pnas.2208998120

or farther from criticality

https://journals.aps.org/prx/abstract/10.1103/PhysRevX.14.031021

In general, explicitly connecting iSTTC-derived timescales to features like cortico-cortical connectivity profiles, laminar-specific circuitry, and mechanisms of dendritic integration would firmly embed the method within a dominant theoretical framework and suggest specific biological mechanisms for the observed differences.

We have now added a new panel (Supp. Figure 12D) in which cortical areas are explicitly ordered along the x-axis according to their hierarchical index, and show that iSTTC-derived timescales broadly recapitulate the findings obtained with more traditional estimators such as PearsonR and ACF. We also added a sentence in the Introduction briefly integrating the studies highlighted by the reviewer that link firing rate and dendritic morphology to intrinsic timescales, to better connect our methodological results to known physiological determinants (lines 21 - 23, see also answer to comment #6 here below).

At the same time, we find that a full mechanistic treatment of how specific biological substrates shape ITs, and how these relate to broader concepts such as criticality, would go beyond the scope of this purely methodological manuscript. We now explicitly state this in the Introduction and refer interested readers to several recent reviews that provide a more comprehensive overview of these theoretical and mechanistic frameworks (see also answer to comment #6 here below).

6. The simulations convincingly show that firing rate affects estimation error, but this could be more deeply contextualized in relation to previous work showing that firing rate and dendritic morphology modulate timescales mechanistically. Integrating these findings could reinforce the idea that timescales reflect both statistical and physiological processes.

<https://doi.org/10.3389/fncel.2024.1404605>

We refered to these findings and corresponding paper in the Introduction, specifying that the firing rate and dendritic morphology can mechanistically modulate intrinsic timescales (line 21). We also refer the interested reader to several recent reviews that discuss this topic in greater detail (lines 22 - 23).

Minor Points:

1. Guidance for Trial-Based Designs: The paper effectively critiques trial-based approaches but does not provide practical guidance for experimentalists. To maximize its utility, the authors should discuss the conditions under which iSTTC remains reliable in a trial-based setting (e.g., minimum number of trials, trial length, and firing rate thresholds) and clarify when continuous recordings are truly essential.

We agree with the reviewer that providing practical guidance for trial-based designs increases the utility of our work. While we cannot propose hard, universally applicable thresholds, our simulations and analyses do identify clear regimes in which IT estimation on epoched data becomes less error-prone. In particular, we show in the manuscript that estimation error decreases with increasing number of trials and with longer trial durations, and that these factors are more critical than firing-rate. We clarify these aspects in section 2.4 and in the third paragraph of the Discussion.

2. Capturing Multi-Scale Dynamics: The analysis assumes neuronal timescales are well-described by a single exponential. It is unclear whether iSTTC can adequately capture the dynamics of neurons with multiple intrinsic timescales. The validity of this assumption should be addressed, potentially by testing alternative models (e.g., double exponentials or power laws) on suitable data.

IT estimation from SUA comprises two steps: (1) construction of an ACF-like function and (2) fitting an exponential to this function. iSTTC only targets step 1 by providing a more robust and less biased estimate of the ACF-like; it does not impose or assume that the underlying dynamics are governed by a single exponential. The detection of multiple intrinsic timescales is handled in step 2 by the chosen fitting procedure. Methods such as aABC are specifically designed to optimize this second step and can, in principle, detect multiple taus. In the revised manuscript, we have added Section 2.7 and Supp. Figure 11 to more directly compare iSTTC and aABC, clarify that they optimize distinct stages of the IT estimation pipeline, and discuss how they could be combined in future work.

3. Computational Efficiency and Scalability: A comment on the computational efficiency of iSTTC is warranted. For the method to be widely adopted, it is important to demonstrate its scalability to very large datasets (e.g., high-density Neuropixels recordings) without prohibitive computational cost. Please include information on algorithmic complexity and computation time.

We followed the suggestion of the reviewer and added information on algorithmic complexity and computation time. The computational cost of iSTTC is linear in the number of spikes (N): each lag is evaluated in time proportional to N, giving a total complexity proportional to n_lags times N. Because n_lags is fixed by the timescale range and does not grow with recording duration, iSTTC scales linearly with spike count and remains efficient even for large datasets. We also confirmed this empirically by using synthetic datasets with signals of increasing length or with an increasing number of trials. Using synthetic datasets of increasing size, we show that iSTTC is computationally efficient and scales linearly with dataset size. Although on the unsegmented dataset iSTTC is slower than ACF on a per-unit basis, processing large datasets (for example, 100,000 units) remains feasible within reasonable compute time. Using synthetic trial-based datasets of increasing size, we show that iSTTC remains computationally efficient and scales linearly with both the number of units and the number of trials. iSTTC is faster than abcTau and PearsonR on a per-unit basis, and processing large trial-based datasets (for example, 100,000 units) is well within practical timeframes.

We included algorithmic complexity estimation in Section 7.2.2 (iSTTC; lines 575 - 582) and Section 7.2.4 (iSTTC for trials; lines 620 - 628). We added Supp. Figures 3, 6, and 11D, which illustrate the computation time for methods applied to unsegmented and epoched data, respectively.

4. Biological Mechanisms in Discussion: The Discussion would be strengthened by more explicitly framing intrinsic timescales (INTs) not just as statistical constructs but as manifestations of underlying biological mechanisms. Briefly linking the findings to substrates like NMDA receptor kinetics, synaptic time constants, and circuit-level excitation-inhibition balance would better bridge the methodological advance to its physiological relevance.

We highlighted the link between intrinsic timescales and underlying biophysical and circuit mechanisms in Introduction by summarizing how synaptic, cellular and network properties can shape ITs. A more extensive discussion of these mechanisms, however, would go beyond the scope of this methodological work, whose primary focus is on improving IT estimation from spike trains. We now explicitly state this in the Introduction and refer interested readers to the same review mentioned in comment #3 of R2 (lines 22 - 23).

5. Terminology Consistency: The terms “ACF,” “ACF-like,” and “pseudo-autocorrelations” should be used consistently to avoid reader confusion. We recommend standardizing all terms throughout the manuscript.

To be more consistent in section 7.2, we have now corrected the terms.

## R3

Reviewer #3: The manuscript introduces a new method, called iSTTC, to estimate the ACF of single-unit activity reliably. iSTTC is an extension of the previously developed spike time tilling coefficient method used for computing correlations between two different spike trains. The authors did a great job of comparing iSTTC to previous methods on synthetic data with known ground truth, as well as on experimental data. The manuscript is clearly written, and the proposed method could provide significant improvements for estimating timescales from single-unit activity. I have just some comments that may help clarify some aspects of the method and its sensitivity to hyperparameters.

We thank the reviewer for the thoughtful comments and the opportunity to clarify our results and their implications. Overall, the reviewers’ suggestions substantially improved the manuscript.

Major comments:

1. Since the main result of this paper is the proposed method itself, it would be helpful to readers to describe the general steps of the method already in the first section of the Results, before discussing how it compares to previous methods. Moreover, a figure illustrating how iSSTC works could clarify some of the comments I am raising below.

We improved the written and graphical description of iSTTC. We followed the reviewer’s suggestion by expanding the method description in Section 2.1 (lines 93 – 127). In addition, we moved Supp. Figure 1 to the main text (now Figure 2), expanded the iSTTC schematic in Figure 2B, and added a new panel (Figure 2C) illustrating the application of iSTTC to trials. We also included pseudocode descriptions of algorithms in Section 7.2.2 (iSTTC) and Section 7.2.4 (iSTTC for trials).

2. Some details of the method are not clear from the current manuscript. I hope the comments below help to clarify them:

- Authors discuss that iSTTC works without needing to bin the data, but in section 7.2.2 they mention n_lags parameter (the total number of time lags), which seems to be an integer. Without binning, the lags should be computed in continuous time, not as an integer, so it was unclear how this parameter is computed and what its role is.

As recommended, we clearly specified that iSTTC does not perform binning or convert spike trains into spike counts per bin. Instead, all computations operate directly on spike times. The parameter n_lags is not a binning parameter; together with the user-defined lag increment (lag_shift), it specifies the time shifts evaluated when computing the autocorrelation (e.g., 50 ms, 100 ms, 150 ms, ...). For example, to obtain an autocorrelation function defined over the interval 0 - 1000 ms with lag_shift = 50 ms, we set n_lags = 20. Thus, n_lags determines how many such time shifts are examined, not how data is discretized. No binning of the spike train takes place at any stage.

To clarify this issue, we expanded Figure 2B and added a new panel (Figure 2C) that schematically illustrates the method parameters. We also included pseudocode descriptions of the algorithm in Section 7.2.2 (iSTTC) and Section 7.2.4 (iSTTC for trials).

- For the trialed version of iSTTC, how is the length of zero padding selected? For experimental data, should it match the inter-epoch intervals of the actual epoched data? Also, is iSTTC computed between spikes across trials (I think here a figure illustration could be quite helpful).

As suggested, we improved the description of the zero-padding procedure. For epoched data, iSTTC is computed on a linearized version of the spike train obtained by concatenating trials. Because iSTTC compares spike timing within a continuous time window, directly concatenating trials would introduce spurious correlations across trial boundaries. To prevent these artefacts, we insert zero-padding between trials. The padding length must be equal to or greater than the duration of a single trial; this ensures that spikes from adjacent trials do not contribute to the iSTTC calculation. Thus, zero-padding does not need to match the actual inter-epoch intervals in the experimental data, as long as it exceeds the trial duration.

We also clarify that iSTTC is not computed between spikes across trials. For each time lag, we construct lag-shifted and unshifted spike segments within each trial, concatenate the corresponding segments with zero-padding, and then compute the STTC between these concatenated spike trains. This ensures that correlations are computed only within appropriate temporal windows and not across trial boundaries.

To clarify this, we expanded the method description in Section 2.1 (lines 110 – 127), added a new panel (Figure 2C) illustrating the application of iSTTC to trials, including how zero padding is applied and how different padding lengths affect the computation, and included a pseudocode description of the algorithm in Section 7.2.4 (iSTTC for trials).

- In Methods, the authors mention “To estimate the area-level intrinsic timescale, all valid ACFs (those without NaNs) from the area were used to fit a single exponential function.” For this, did the authors average over ACF all neurons and then fit the average ACF, or did they fit a single function to all individual ACFs? In the latter, what is the logic behind having the same timescale for all neurons, given that from many studies we know there is a large diversity across single-neuron timescales within an area?

In our analysis, we fit a single exponential function to all individual ACFs within an area. We also evaluated the approach, in which we first average the ACFs across neurons in the area and then fit an exponential decay to the averaged ACF; both procedures yielded identical time constants. The only differences were in the width of the confidence intervals and the resulting R-squared values. To illustrate these two options, we included the figure below, which shows that, for one brain area (PM) and one method (ACF), fitting a single function across all individual ACFs (left) or fitting the exponential decay to the averaged ACF (right) produces identical time constants.

Revision Figure 2. Line plot displaying the estimated IT by fitting a single function across all individual ACFs (left) or fitting the exponential decay to the averaged ACF (right).

Of note, this area-level fit is not used to claim that all neurons share the same intrinsic timescale. At the unit level, we fit an exponential function to each neuron’s ACF separately and obtain a neuron-specific time constant. This results in a distribution of timescales within each area, which is consistent with the known heterogeneity reported in the literature.

- It would be helpful if the authors show example ACFs and their exponential fits from both methods in Figs 2 and 3, in addition to statistics, to visualize cases where each method is better and also compare them to the ground-truth shape of ACF of the Hawkes process. Also, for Fig 5, it would be helpful to see how the shape of example ACFs changes depending on the method and highlight how it affects their inclusion based on different criteria.

To address the reviewer’s suggestion, we added three new supplementary figures:

Supp. Figure 1 shows representative cases where iSTTC (panel A) or ACF (panel B) performs better on unsegmented signals. For iSTTC, these cases correspond to high excitation strength and low firing rates. For ACF, they correspond to low excitation strength combined with high firing rates. We show the unit ACFs, the fitted exponential decay, the ground-truth autocorrelation of the Hawkes process, and provide examples for both low and high intrinsic timescales.

Supp. Figure 5 presents examples for trial-based data, illustrating conditions under which iSTTC (panel A) or PearsonR (panel B) performs better. iSTTC performs better at high excitation strength. PearsonR performs better at low excitation strength. As above, we include examples for both low and high intrinsic timescales and show the unit ACFs, the fitted exponential decay, and the ground-truth ACF.

Supp. Figure 9 provides examples of exponential fits for all four methods. For each unit, we show the unit ACFs, the fitted exponential decay for four methods, and the ground-truth autocorrelation from the Hawkes process. We also indicate the inclusion criteria to highlight how method-dependent ACF shapes influence which units are retained.

3. The authors discuss that iSTTC method has several hyperparameters, such as n_lags, lag_shift, Delta t, and zero_padding length. For the application of the method, it .would be important to show how the choice of these hyperparameters affects the estimated timescale. Testing for hyperparameter-sensitivity is also important for comparing with classic methods that use bin size as a hyperparameter. The selected hyperparameters need to be mentioned in the figure captions (or as a table).

As recommended, we expanded the method and parameters description, and we also added a new analysis assessing how n_lags and lag_shift affect the results.

For epoched data, iSTTC is applied to a linearized spike train in which trials are concatenated with zero padding. This padding must be at least as long as the trial duration to prevent spurious correlations across trial boundaries. Once this minimum is satisfied, the exact padding length does not affect the estimated timescale. To assess the effect of other parameters, we estimated intrinsic timescales using four methods. For all methods, we computed the autocorrelation function over the interval 0-1000 ms. For ACF and PearsonR, which operate on binned data, we varied the bin_size parameter using values of 10, 40, 50, 60, and 100 ms. For iSTTC, we used the same set of values as the lag_shift parameter, with corresponding n_lags values of 100, 25, 20, 16, and 10. We show that the choice of bin_size (or lag_shift for iSTTC) affects the number of rejected units, with the highest rejection rates occurring at the smallest bin size/lag_shift. Bin size/lag_shift also influences the estimated intrinsic timescale, although the effect is inconsistent across bin sizes/lag_shifts: at 10 ms the effect is strongly negative, at 40 and 100 ms it is negligible, and at 60 ms it becomes slightly positive. These patterns are largely similar across methods.

We updated the explanation in Section 2.1 (lines 93 – 127), expanded the iSTTC schematic in Figure 2B, and added Figure 2C illustrating how padding is applied and how different padding lengths influence the computation. We also included pseudocode descriptions of algorithms in Section 7.2.2 (iSTTC) and Section 7.2.4 (iSTTC for trials). We added Supp. Figure 8, which illustrates the effects of n_lags and lag_shift on intrinsic timescale estimation, and a new panel C to Supp. Figure 10, which illustrates the effects of n_lags and lag_shift on the number of rejected units. We also added parameters to the figure captions (Fig.3, Fig.4, Fig.5, Fig.7).

Minor comments:

- This sentence, “In conclusion, also on experimental data, iSTTC outperforms ACF and PearsonR IT estimation accuracy.”, is not formulated quite accurately, since for the experimental data, there is no ground truth to test which method outperforms which.

We modified the title of the section containing that sentence, the highlighted sentence (line 446 - 447) and explicitly address the limitations of IT estimation accuracy comparisons on experimental data in the Discussion (lines 456 - 459). See also reply #1 to R2.

- The authors use “continuous data” to refer to long trials/time-series, but the term is a bit misleading, especially in the abstract, since it might convey that iSTTC can be applied to continuous signals such as LFP.

We have replaced the term “continuous data” with “unsegmented” throughout the manuscript.
