# Supplementary material for: iSTTC: A robust method for accurate estimation of intrinsic neural timescales from single-unit recordings
Source: PLoS Comput Biol. 2026 Mar 16;22(3):e1013385. doi: 10.1371/journal.pcbi.1013385 (PMC13012623; doi:10.1371/journal.pcbi.1013385)
Supplement: Attachment — Submitted filename: response_to_reviewers_auresp_2.docx [file pcbi.1013385.s016.docx]

# **Reviewer #1:**

I thank the authors for their detailed response. In combination with the changes in the manuscript, all my concerns are very well addressed. I recommend publication after addressing the minor comments (typos etc.) below.

In my opinion, the revision substantially improved the manuscript. In particular, the extended introduction of the method in Section 2.1 and Figure 2 make it much easier to understand the method - including the implementation of trial concatenation. The detailed comparison to adaptive Approximate Bayesian Computation roots the work nicely in the literature (or, as another reviewer put it, the methodological ecosystem) and highlights its strengths. Finally, I very much appreciate the additional discussions regarding fundamental limits in the estimation of intrinsic timescales.

We thank the reviewer for the constructive feedback throughout the review process and for the positive assessment of the revised manuscript.

Minor comments:
* Typesetting in Eq. (1): Delta and t should both be in the subscript

We fixed the typesetting.

* Fig. 2 caption: what is t (presumably a Delta is missing)?

We changed t to delta_t in the caption of Fig. 2.

* The supplemental figures in the zip file seem mixed up (e.g., Supp. Fig. 11 seems to be SuppFigure8.tiff)

We corrected the file names of the supplementary figures.

Beyond the scope of this review process, I am very curious if there is a direct mathematical link between iSTTC and the more traditional ACF estimates. For example, do they converge to the same value for infinite data? If there is such a link, adding it to the supplement would in my opinion significantly strengthen the underlying theoretical foundations of the method (and also provide some insight on the unification of measures discussed in response to reviewer 2, point 3). However, in my opinion it should be fully at the authors' discretion whether or not to include this; the empirical evidence in the manuscript provides a clear

picture.

We thank the reviewer for this suggestion and fully agree that establishing a formal mathematical link between iSTTC and classical ACF estimators would be valuable. However, developing such a result would require a dedicated theoretical analysis and goes beyond the scope of the present study, which focuses on empirical validation. We consider this an important direction for future work, ideally in a follow-up study that systematically compares intrinsic timescale estimators (on both continuous and discrete data) and clarifies their relationships.

As a first step in this direction, we have included a dedicated question on intrinsic timescales within the CON2PHYS project (https://mchini.github.io/con2phys/), a community-driven initiative that quantifies the degree of consensus among neuroscientists in interpreting fundamental concepts and analyses derived from in vivo electrophysiological data. This will allow us to obtain a systematic overview of the methods currently used to estimate intrinsic timescales and to evaluate how consistent the resulting estimates are across approaches. We hope that this effort will provide an empirical foundation for a more comprehensive theoretical and methodological comparison of timescale measures in future work.

**Reviewer #2:**

All my concerns have been addressed, and I recommend publication.

We thank the reviewer for the constructive feedback throughout the review process and for the positive assessment of the revised manuscript.

# **Reviewer #3:**

I thank the authors for considering my comments and carefully addressing them, which improved the clarity of the manuscript. I have a few remaining comments on the revised manuscript:

We thank the reviewer for their careful re-evaluation of the manuscript and for the constructive feedback, which has helped improve its clarity.

1. I am confused by Supp. Fig 1 and 5, how come the ground-truth autocorrelation (black line) is sometimes different between iSTTC and ACF panels (e.g., the first row of panel A in Supp. Fig 1)? The ground-truth autocorrelation should be independent of how the data autocorrelation is estimated (by iSTTC or ACF).

We agree with the reviewer that the current visualization is confusing. The ground-truth autocorrelation is indeed independent of the method used to estimate the empirical autocorrelation and is the same for both iSTTC and ACF. The discrepancy in the figures arises from the normalization used for visualization. Specifically, in Supp. Figs. 1, 5, and 9, the black curve represents the analytical ground-truth autocorrelation ACFtrue(t) = exp(−t/tau), which is identical across panels. However, for display purposes, this analytical ACF is scaled separately in each panel to match the amplitude of the corresponding empirical estimate (iSTTC or ACF) at the first non-zero time lag:

ACFtrue, scaled(t) = ACFemp(lag_1) / ACFtrue(lag_1) * ACFtrue(t)

where lag_1 is the first non-zero lag, and ACFemp is either the iSTTC or ACF estimate.

Because iSTTC and ACF estimates differ in their intrinsic normalization and amplitude scaling, the resulting scaled ground-truth curves differ slightly between panels, even though they originate from the same analytical function. This normalization was chosen to facilitate shape-based comparison between empirical estimates and the ground truth. Without this normalization, differences in amplitude would dominate the visual comparison and obscure discrepancies in decay rate (Revision Figure 1). Importantly, this normalization does not affect the estimation of the time constant, which depends on the decay shape rather than absolute magnitude.

To improve clarity, we now explicitly state the applied scaling in the captions of Supp. Figures 1, 5, and 9.

Revision Figure 1. Representative examples of autocorrelation functions and exponential fits (from Supp. Figure 1B, bottom left). **(A)** Visualization with scaling of the ground-truth autocorrelation. **(B)** Same data without scaling, illustrating how amplitude differences dominate the visual comparison.

2. The filenames of some of the attached tif files of supplementary figures do not match their captions/descriptions in the text. For instance, the Supp. Fig 9 tif file is Supp. Fig 8, Supp. Fig 10 tif file is probably Supp. Fig 11, etc. At least for reviewing, it would have been helpful if the supplementary figures were included in the manuscript together with their captions.

We corrected the file names of the supplementary figures. We share the reviewer’s view that presenting figures together with their captions is clearer for the reader, but this was not possible in light of the journal’s formatting guidelines for revised papers.

3. A few comments about the new section comparing aABC and iSTTC:

- The authors mentioned (line 338) “aABC is a method that improves IT estimation by optimizing the exponential fitting of the ACF”; this is not accurate because the aABC method does not fit exponential functions at all; it replaces the exponential fits by comparing summary statistics of synthetic data from a generative model with the real data.

To address the reviewer’s comment, we changed the phrasing to “aABC is a method that improves IT estimation by optimizing the *step of extracting timescales based on the autocorrelation function*, leveraging generative models to simulate synthetic signals across a range of candidate timescales, compute their autocorrelation functions, and return a posterior distribution over timescales.” We also replaced “exponential fitting” with “timescale extraction” in lines 353, 385, and 386.

- I completely agree with the authors that these two methods are addressing two complementary problems, ACF estimation versus timescale estimation, and in principle, their combination could improve the overall process. At the same time, choosing the right summary statistics is an important step for all simulation-based inference methods, including ABC or SBI; therefore, it is not quite right to compare aABC with iSTTC when the summary statistics are not matched. Considering this point, it is misleading to suggest aABC is not suitable for SUA data because of its higher rejection rate. If authors use iSTTC as the summary statistic of aABC, the rejection rates will probably be comparable, but I agree that this is beyond the scope of this manuscript. My suggestion is that authors revise this section to clarify the difference in the summary statistics between methods and the fact that with better summary statistics, aABC will probably have comparable results for epoched SUA data.

We agree with the reviewer that the performance of aABC depends on the choice of summary statistics and that directly comparing methods without explicitly stating these choices can be misleading. We have now clarified this in the manuscript and explicitly noted that alternative summary statistics could affect both rejection rates and estimation accuracy (lines 349-351, 365-367, 382).

- Of course, it is fair to say that aABC method has a higher computational cost because it uses simulations from generative models instead of exponential fitting. However, again, the speed of the simulation-based methods depends on the chosen summary statistics and the generative model. Based on the toolbox description (https://github.com/roxana-zeraati/abcTau), it seems the authors have chosen the slowest summary statistics “comp_cc” which computes Pearson correlations in the time domain, but presumably this step could be significantly sped up if the authors used an FFT-based method (Although it would still be slower than exponential fitting, the differences may be smaller). This point needs to be clarified in the manuscript.

To address the reviewer’s comment, we benchmarked the “comp_cc” summary statistic against the FFT-based autocorrelation calculation provided in the abcTau toolbox (“comp_ac_fft”) using a synthetic dataset of epoched data. As expected, the FFT-based approach substantially reduces the computational cost of the summary statistics step. On average, comp_ac_fft was approximately 13.6-fold faster than comp_cc (0.00012 s vs. 0.00168 s per unit), corresponding to a ~92.6% reduction in runtime. If we assume that the dominant computational cost in aABC arises from repeated summary-statistics calculations, then replacing the time-domain implementation with an FFT-based one would reduce the median runtime per unit from 211 s to approximately 15.5 s. Even under this assumption, aABC would remain roughly 250–400 times slower than PearsonR (0.06 s) and iSTTC (0.04 s), respectively.

We have clarified this in the manuscript (lines 373-377).

- and of course, as the authors mentioned, for long time series, aABC does not add much given that the ACF bias is negligible. So it does not make sense to use aABC for unsegmented data with long durations if there is no bias.

We agree with the reviewer and mention this in the manuscript (lines 347–349).

4. Fig 2C caption needs a bit more detailed description. For example, how is the intrinsic timescale difference calculated? Is the difference from the ground truth? If yes, the parameters of the process/ground truth need to be specified.

To address the reviewer’s comment, we clarified in the caption of Fig. 2C that the intrinsic timescale difference is computed relative to the reference condition in which the zero-padding length equals the trial length T. Thus, the estimate obtained with padding length T serves as the baseline for comparison, rather than the generative ground-truth timescale.

5. There is a typo in equation 1: it should be STTC_{\Delta t}

We fixed the typesetting.

6. There is a mismatch between the notation that is used in Figure 2, its caption, and the main text description. The time lag in the main text and figure is \Delta t, but in the figure caption, it is t.

We changed t to delta_t in the caption of Fig. 2.
